# Perceptual restoration of masked speech in human cortex

**DOI:** 10.1038/ncomms13619

**Published:** 2016-12-20

**Authors:** Matthew K. Leonard, Maxime O. Baud, Matthias J. Sjerps, Edward F. Chang

**Affiliations:** 1 Department of Neurological Surgery, University of California, San Francisco, 675 Nelson Rising Lane, Room 535, San Francisco, California 94158, USA; 2 Center for Integrative Neuroscience, University of California, San Francisco, 675 Nelson Rising Lane, Room 535, San Francisco, California 94158, USA; 3 Department of Neurology, University of California, San Francisco, 675 Nelson Rising Lane, Room 535, San Francisco, California 94158, USA; 4 Department of Linguistics, University of California, Berkeley, 1203 Dwinelle Hall #2650, Berkeley, California 94720-2650, USA; 5 Neurobiology of Language Department, Donders Institute for Brain, Cognition and Behavior, Centre for Cognitive Neuroimaging, Radboud University, Kapittelweg 29, Nijmegen 6525 EN, The Netherlands; 6 Department of Physiology, University of California, San Francisco, 675 Nelson Rising Lane, Room 535, San Francisco, California 94158, USA

## Abstract

Humans are adept at understanding speech despite the fact that our natural listening environment is often filled with interference. An example of this capacity is phoneme restoration, in which part of a word is completely replaced by noise, yet listeners report hearing the whole word. The neurological basis for this unconscious fill-in phenomenon is unknown, despite being a fundamental characteristic of human hearing. Here, using direct cortical recordings in humans, we demonstrate that missing speech is restored at the acoustic-phonetic level in bilateral auditory cortex, in real-time. This restoration is preceded by specific neural activity patterns in a separate language area, left frontal cortex, which predicts the word that participants later report hearing. These results demonstrate that during speech perception, missing acoustic content is synthesized online from the integration of incoming sensory cues and the internal neural dynamics that bias word-level expectation and prediction.

## Abstract (web summary)

We can often ‘fill in' missing or occluded sounds from a speech signal—an effect known as phoneme restoration. Leonard *et al*. found a real-time restoration of the missing sounds in the superior temporal auditory cortex in humans. Interestingly, neural activity in frontal regions prior to the stimulus can predict the word that the participant would later hear.

## Graphical abstract

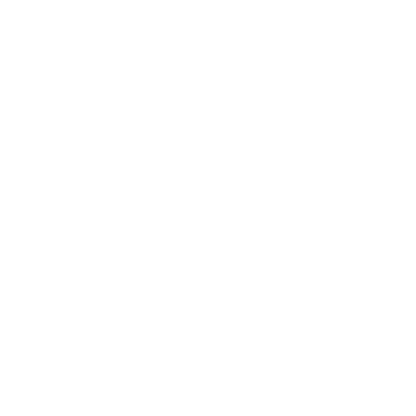

Spoken communication routinely occurs in noisy environments^1^ such as restaurants, busy streets and crowded rooms, making it critical that the brain can either reconstruct or infer the sounds that are masked^2^,^3^. Often, the perceptual system ‘fills in' those obscured sounds^4^ without the listener's awareness^5^. This continuity effect is a crucial feature of speech perception, where relevant contrastive sounds (phonemes) last only a few hundred milliseconds, on the same temporal scale as many extraneous masking sounds (for example, clattering dishes and car horns). The brain mechanisms that give rise to this striking perceptual experience known as phoneme restoration are unclear.

To examine the neural basis of perceptual restoration, we developed a set of stimuli that differed in a single critical phoneme (‘original'; for example, ‘fa***s***ter' [/fæstr/] versus ‘fa***c***tor' [/fæktr/]; Fig. 1a,b, other examples include: ‘no***v***el' versus ‘no***zz***le', 'rigi***d***' versus ridge***s***', '***b***abies' versus ‘***r***abies': Supplementary Fig. 1, see Supplementary Table 1 for all examples), following work by Samuel^6^. Auditory neural populations on the human superior temporal gyrus (STG) discriminate between speech sounds^7^, allowing us to compare responses to each of the two original stimuli. To evoke perceptual restoration, participants also heard stimuli that had the critical phoneme completely replaced with broadband noise (‘noise'; /fæ***#***tr/, /n>***#***=l/, /rFdW=***#***/, /***#***ebiz/; Fig. 1c). On each trial, participants reported which of the two original words they heard. Listeners subjectively heard one word or the other (not both) on each presentation of a given noise stimulus (Fig. 1d; see also the Methods section for a description of the behavioural pilot study). As in natural listening conditions, many factors can influence whether a listener will experience perceptual restoration, and it is even more difficult to know *a priori* whether a particular stimulus will be perceived in a bistable manner on separate trials. Despite these challenges, we succeeded in identifying a set of stimuli where each participant perceived the identical noise stimulus as both possible original words. To explicitly bias participants to hear the noise stimuli as specific words, a subset of patients heard the stimuli following sentence frames (for example, ‘He went to the bookstore to buy the [/n>***#***=l/]'; see the ‘Methods' section; examples of sentence-biased data are presented in Supplementary Figs 1 and 2).

While participants listened to these stimuli, we recorded direct cortical activity from a high-density multi-electrode electrocorticography (ECoG) array implanted for the clinical purpose of seizure localization. Direct neural recordings possess excellent spatial and temporal resolution with a high signal-to-noise ratio, allowing the detection of speech signals at the level of individual phonetic features^7^. These properties offer a powerful method to address the cortical representation of subjective perception on a single-trial basis^8^. The task and stimuli allowed us to examine whether neural responses to noise were more, or less, similar to the perceived phoneme on individual presentations. Specifically, we asked whether the speech auditory cortex generates representations of the missing phonemes in real time.

## Results

### Single electrode restoration effects

**Figure 1:**
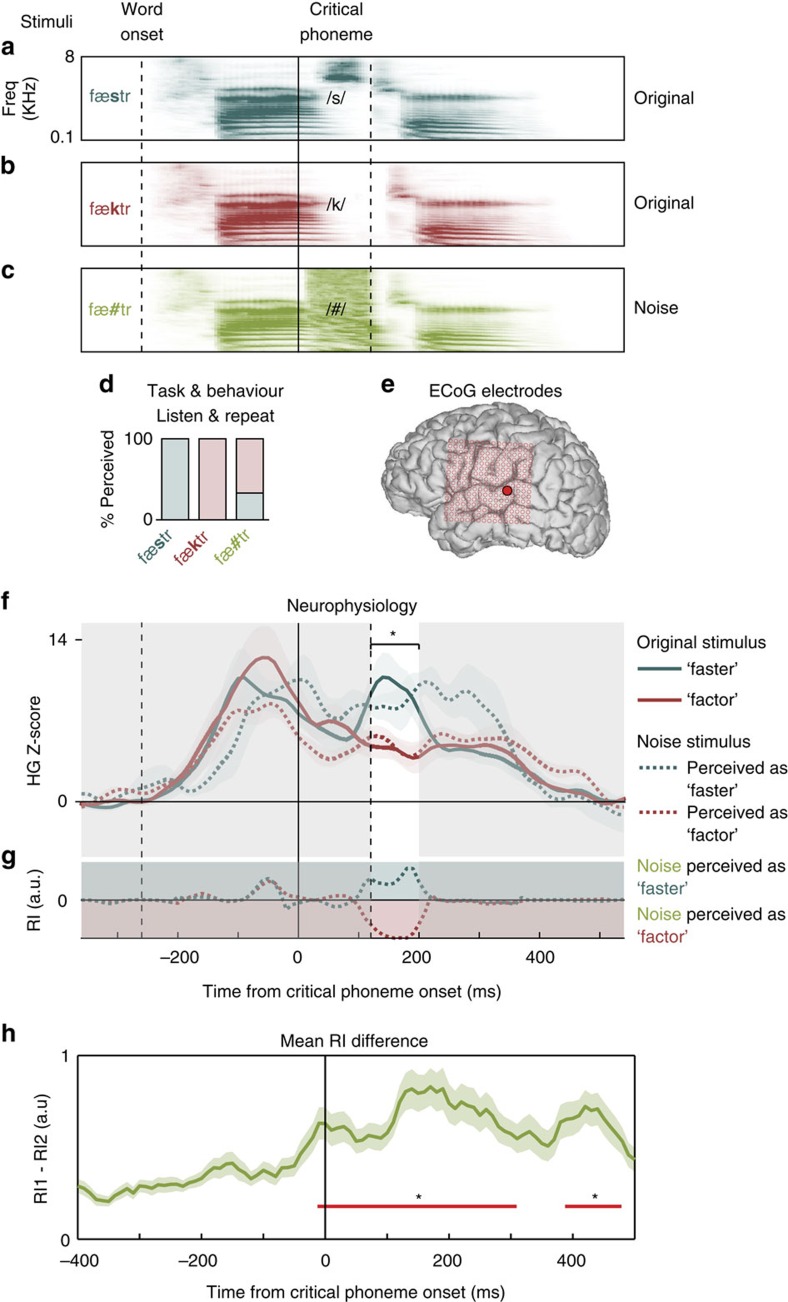
Stimuli and single electrode online phoneme restoration effects. (**a**,**b**) Participants listened to pairs of spoken words (/fæ**s**tr/ (**a**) versus/fæ**k**tr/ (**b**)) that were acoustically identical except for a critical phoneme that differentiated their meaning (vertical solid and second dashed lines; first dashed line is word onset). (**c**) The critical phoneme was also replaced by broadband noise (/fæ**#**tr/), and on each trial, participants reported which word they heard. (**d**) Behavioural results show bistable perception on noise trials. (**e**) Location of representative posterior STG electrode in **f**. (**f**) STG electrode shows selectivity for /s/ compared to /k/ (solid blue line stronger response than solid red line immediately after critical phoneme, unshaded region). Trials were sorted depending on which word participants perceived. Responses to noise stimuli were similar to the original version of the perceived phoneme (dotted lines; *signifies 99% CIs only overlapping for same coloured curves; shaded error±s.e.m. across trials). (**g**) RI describes the magnitude of neural restoration as the relative distances between each noise and original pair in **f**. When the dotted line is in the region shaded with the same colour, the electrode's activity reflects the participant's percept. (**h**) Across all participants, word pairs and electrodes, the magnitude of the difference between RI values illustrates that when these neural populations differentiate original stimuli, they also differentiate noise trials, beginning at the onset of the critical phoneme (red bar, one-way *t* tests, *P*<0.05, Bonferroni corrected). Shaded error±s.e.m. across word pairs.

To evaluate whether cortical responses to noise stimuli (for example, /fæ#tr/) were similar to responses to the original speech sounds, we examined electrodes that discriminated the two original phonemes. For example, an electrode over left non-primary auditory cortex (STG; Fig. 1e; Supplementary Fig. 1) showed a larger high-gamma response (increased power at 70–150 Hz) to /s/ compared with /k/ beginning ∼100 ms after the onset of the critical phoneme (99% CIs not overlapping; Fig. 1f, solid lines). Therefore, this electrode differentiates ‘faster' and ‘factor' by encoding the difference between fricative (/s/) and plosive (/k/) speech features ∼100 ms after the onset of the critical phoneme^7^,^9^.

Next, the noise stimulus trials were sorted according to which word the participant reported hearing. When /fæ#tr/ was perceived as /fæstr/, the electrode showed a stronger response than when the stimulus was perceived as /fæktr/ (99% CIs not overlapping; Fig. 1f, dashed lines). Critically, during the time window of maximal neural discriminability between the two original stimuli, the neural responses to the noise stimulus closely tracked the neural responses to the original stimuli (∼120–240 ms after critical phoneme onset, consistent with typical STG response latencies, 99% CIs overlapping for the perceived phoneme, but not the other phoneme, Fig. 1f). We did not observe systematic differences in the nature of the online restoration effect when the stimuli were embedded in sentence contexts (Supplementary Fig. 1), therefore we combined the data from the single word and sentence tasks. However, we present example effects from both tasks in the following sections.

To quantify the magnitude of this effect, we computed a restoration index (RI) for each electrode, which reflects the distance between each noise response and each original response. During the auditory response time epoch immediately following the critical phoneme, the RI showed that responses to noise were more similar to the perceived phoneme (an example for the single electrode is shown in Fig. 1g). Across all participants, word pairs, and electrodes (Supplementary Fig. 3), significant neural phoneme restoration effects began around the onset of the critical phoneme. The absolute value of the difference between RI time-courses shows that neural populations discriminate between the two perceptions of the noise stimulus with similar timing as they do between the two perceptions of the two original stimuli (Fig. 1h). Neural phoneme restoration was strongest ∼150 ms after critical phoneme onset (one-sample *t* test across 131 electrodes *P*<0.05, false discovery rate corrected for 91 time points), consistent with the relatively short latency encoding of acoustic information^9^, and consistent with online warping of noise to phoneme percepts.

**Figure 2:**
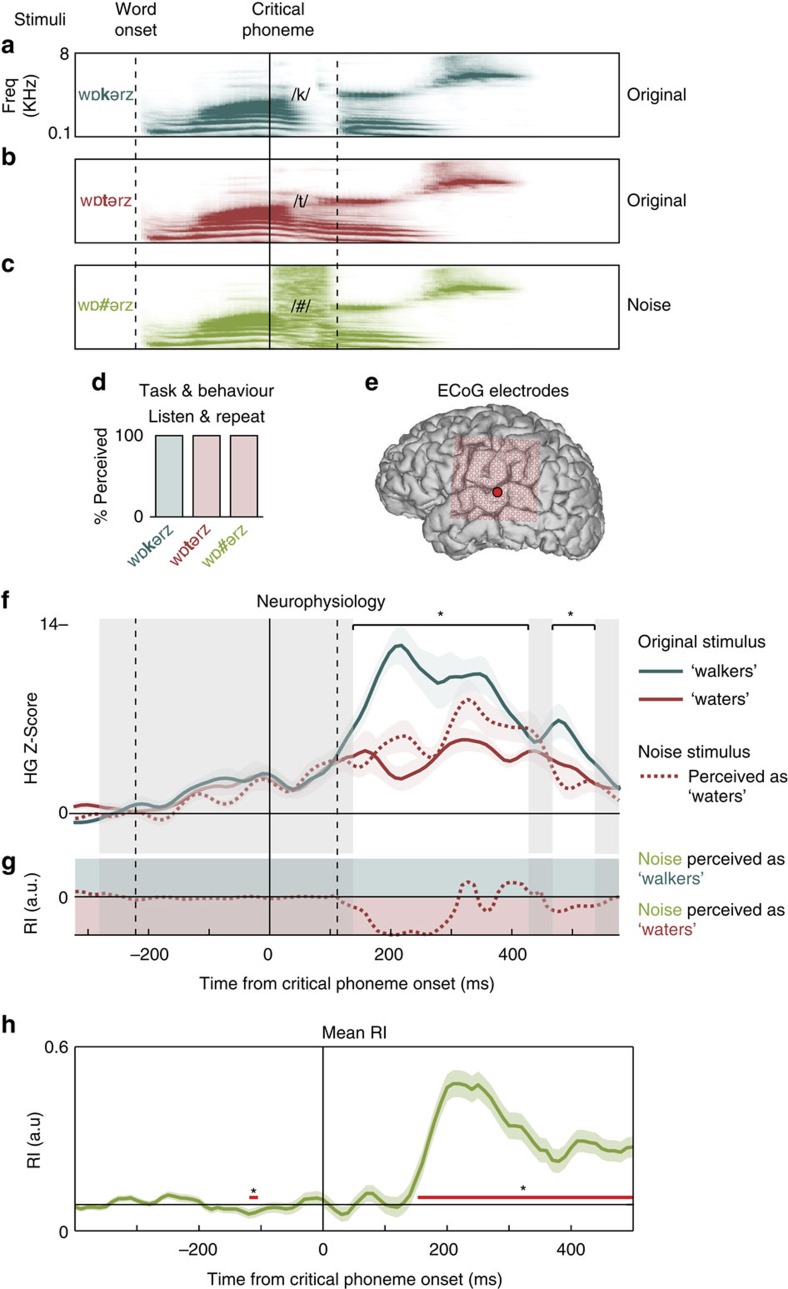
Stimuli and single electrode online phoneme restoration effects for a representative word pair where the participant did not show bistable perception. (**a**,**b**) Subjects listened to pairs of spoken words (/wƅ**k**ǝrz/ (**a**) versus/ wƅ**t**ǝrz/ (**b**)) that were acoustically identical except for a critical phoneme that differentiated their meaning (vertical solid and second dashed lines; first dashed line is word onset). (**c**) The critical phoneme was also replaced by broadband noise (/wƅ**#**ǝrz/), and on each trial subjects reported which word they heard. (**d**) Behavioural results showed that the noise was always perceived as /t/. (**e**) Location of representative STG electrode in **f**. Data are from the same subject as in Fig. 1. (**f**) Single representative left hemisphere STG electrode shows selectivity for /k/ compared with /t/ (solid blue line stronger response than solid red line immediately after critical phoneme, unshaded region). Responses to noise stimuli were similar to the original version of /t/ (dotted red line; *signifies 99% CIs only overlapping for red curves; shaded error±s.e.m. across trials). (**g**) RI describes the magnitude of neural restoration as the relative distances between each noise and original pair in **f**. When the dotted line is in the region shaded with the same colour, the electrode's activity reflects the subject's percept. (**h**) Across all word pairs that did not exhibit bistable perception, the average timecourse of the RI metric for all electrodes shows neural restoration effects beginning ∼150 ms after critical phoneme onset (red bar: one-way *t* test against baseline, *P*<0.05, false discovery rate corrected for time points). Shaded error±s.e.m. across word pairs.

As discussed above, it is difficult to predict whether and when listeners will experience bistable perceptual restoration on a given stimulus. Therefore, we presented participants with many word pairs to maximize the chances of finding bistable effects. In addition to our analysis of the bistable stimuli, the subset of stimuli for which bistable perception did not occur can still be used to characterize the similarity in neural processing between the noise stimulus and each of the two originals. This can provide additional confirmation of the online neural restoration effect. For example, one participant always heard /w3**#**=rz/ as ‘waters', and not ‘walkers' (Fig. 2a–e). On electrodes that discriminated the two original phonemes /t/ and /k/, neural responses to noise closely tracked neural responses to the original phoneme that was perceived (Fig. 2f,g). Across all stimuli that did not elicit bistable perception, the mean RI illustrated the online restoration effect beginning ∼150 ms after critical phoneme onset (Fig. 2h; one-sample *t* test across 920 electrodes *P*<0.05, false discovery rate corrected for 91 time points), consistent with stimuli that elicited bistable perception.

These results confirm the general timing of neural restoration that we observed in the important cases of bistable perception. However, it is difficult to rule out the influences of various acoustic and perceptual factors in biasing listeners to hear the noise as only one sound. For example, it is known that the acoustic similarity between noise and the perceived phoneme influences the strength of perceptual restoration^2^,^10^, as do coarticulatory cues in the preceding speech sounds (which were mostly excised from these stimuli, but could still be present in subtle forms). Therefore, to allow us to examine the mechanisms of perceptual restoration, we focused further analyses on stimuli where participants heard the same physical noise stimulus as both possible phonemes.

### Stimulus spectrogram reconstruction

**Figure 3:**
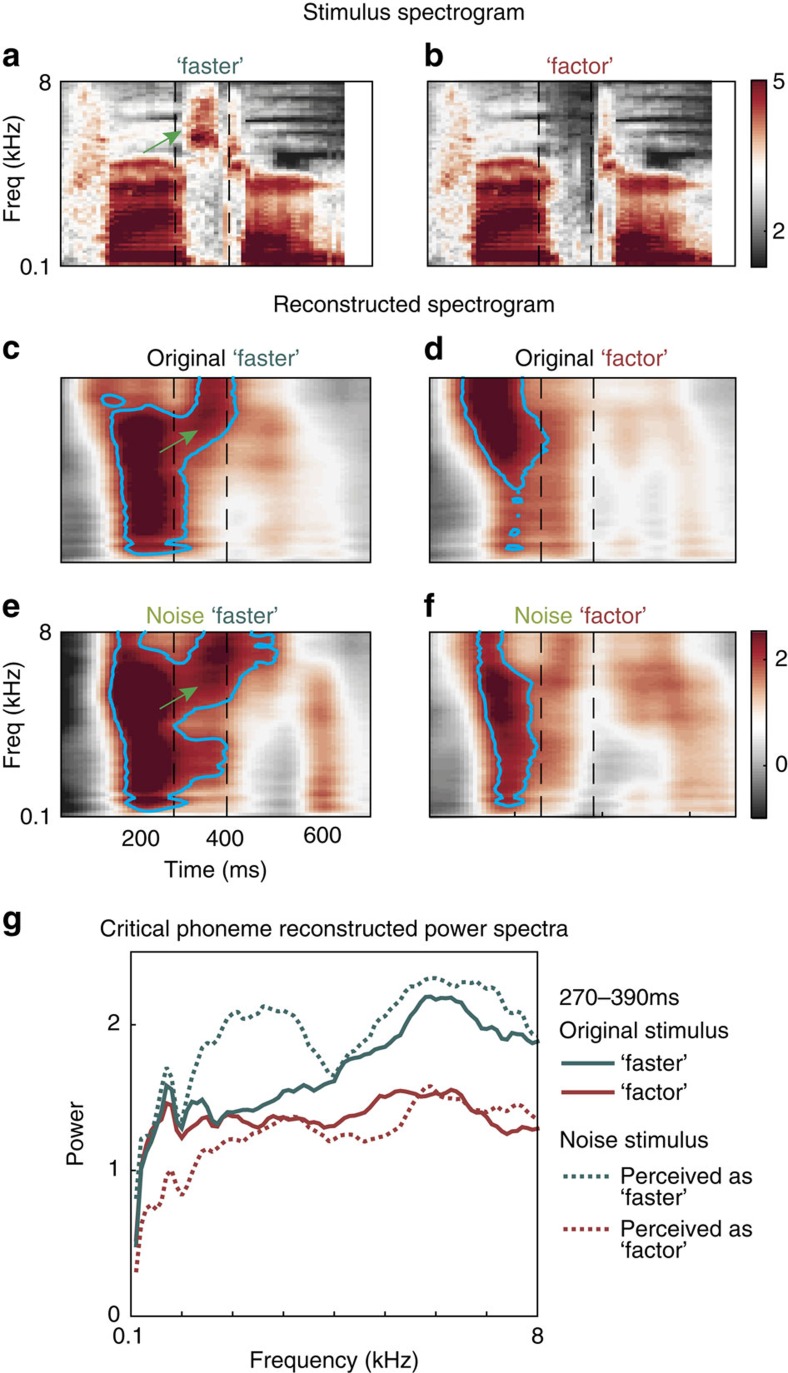
Stimulus spectrogram reconstruction reveals warping of noise to perceived phoneme. (**a**,**b**) Acoustic spectrograms for a representative word pair (/fæstr/, (**a**), versus /fæktr/, (**b**)) differ primarily in the presence of a high-frequency component during the critical phoneme in **a** (green arrow). (**c**,**d**) Spectrograms from (**a**,**b**) reconstructed from electrode population activity show that the high-frequency component is present in / fæstr/ (**c**, green arrow) and absent in /fæktr/ (**d**). (**e**,**f**) Spectrogram reconstruction of noise trials was divided according to which word the participant heard on each trial. During the critical phoneme, a high-frequency component is visible only for trials perceived as /fæstr/ (**e**, green arrow) and not for /fæktr/ (**f**). (**g**) Power spectra of the critical phoneme for **c**–**f** show close correspondence between noise and original phonemes, particularly in mid-high frequencies.

We confirmed these single electrode results across the electrode population using stimulus spectrogram reconstruction^11^, a linear decoding method for examining how electrode population neural responses encode the fine-scale spectrotemporal features of acoustic input. The stimulus reconstruction filters were created using neural responses to a natural speech corpus. In the example pair, ‘faster' versus ‘factor', the two original spectrograms are distinguished primarily by a high-frequency frication component during the critical phoneme (Fig. 3a,b, green arrow). The neural reconstructions of the original stimulus spectrograms clearly reflected the fricative in ‘faster', which was absent for ‘factor' (Fig. 3c,d, green arrow). When the spectrogram of the noise stimulus (/fæ#tr/) was reconstructed separately for each percept, the primary distinction that again emerged was the high-frequency component when the noise was perceived as /s/ but not /k/ (Fig. 3e,f, green arrow).

Across all word pairs and participants, the mean correlation between the original and noise reconstructions of a perceived phoneme was higher compared with the correlation between the reconstruction of an original phoneme and the other noise percept (*r*=0.58 versus *r*=0.37). In particular, the power spectrum of the critical phoneme was closely matched for original and restored versions of the same sound (Fig. 3g; see also Supplementary Fig. 2 for an example from the sentence task; Mann–Whitney *U* test on Euclidean distances between critical phoneme spectra for all word pairs in frequencies that maximally discriminate originals, *z*=−2.14, *P*=0.03, *n*=24).

To confirm that reconstruction of spectrotemporal features is driven by auditory cortex, we performed the analyses separately for STG and frontal electrodes, regardless of whether individual electrodes in these areas discriminated between the two original phonemes. We found that for STG electrodes, reconstruction of the critical spectrotemporal distinction between /k/ and /s/ (high-frequency frication) is apparent for both original and noise ‘faster', but not for ‘factor' (Supplementary Fig. 4c–f). These results are highly consistent with what we observed using all electrodes that discriminate original /k/ and /s/ (Fig. 3), and in fact slightly improve the fidelity of the reconstruction, particularly in low frequencies. In contrast, reconstruction using only frontal electrodes was poor, and did not represent any distinctive features of the stimulus spectrograms (Supplementary Fig. 4g–j). We observed the same pattern for ‘novel' and ‘nozzle' in the sentence task, where the high-frequency reconstruction is driven entirely by STG electrodes (Supplementary Fig. 5). These results demonstrate that auditory neural population responses to restored phonemes reflect a processing bias of the noise burst towards the spectrotemporal acoustic features of the perceived sound.

### Pre-stimulus bias effects

It is remarkable that auditory cortex processes the noise input as distinctive phonetic information so rapidly. To understand how online restoration is possible, we performed several additional analyses. Behaviourally, for items presented in the single word paradigm, listeners were more likely to hear whichever original word in the pair they heard previously during the task (mean=75.8%±21.1% of trials). For example, in the sequence of trials, if they heard ‘faster' before a noise trial, /fæ#tr/, they were more likely to report the noise as ‘faster'. This suggests that rapid online restoration is at least partially related to perceptual, lexical and/or semantic priming^12^,^13^,^14^.

In addition to priming effects, there is growing evidence that stochastic fluctuations in ongoing cortical activation can influence sensory and perceptual processing^15^. These fluctuations may be task-irrelevant, but can reflect attractor states that alter perception. To explore how such rapid processing of noise as phonemic percepts is possible, we leveraged single-trial analyses of neural population dynamics^16^. We visualized brain states using principal component analysis (PCA) to represent activity across electrodes covering both auditory and non-auditory areas over time as trajectories through a lower-dimensional ‘neural state-space'. This qualitative analysis provides an opportunity to examine the temporal evolution of activity through the network without imposing anatomical priors on which brain regions should contribute to different effects. The first two principal components (accounting for 47.1% of the variance across electrodes; mean±s.d. across subjects=39.4±12.2%; Supplementary Fig. 6) clearly illustrated that the population activity discriminated both original and noise trials according to the participant's perception beginning around the middle of the critical phoneme (Supplementary Fig. 7, blue arrow; Supplementary Fig. 8).

**Figure 4:**
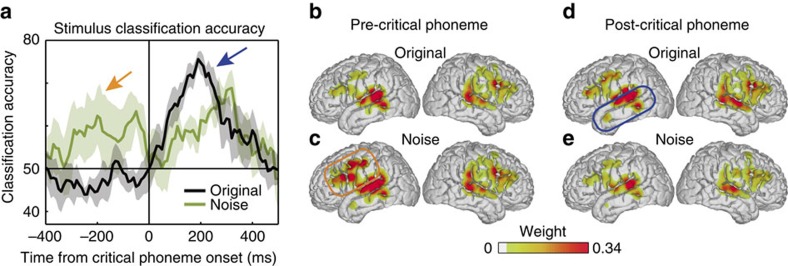
Timecourse of stimulus classification shows pre-stimulus frontal lobe bias for restored phonemes. (**a**) Trials were classified using population neural activity and compared with reported perception. Original word classification accuracy peaked ∼200 ms after critical phoneme onset (black line, blue arrow). Noise trial classification accuracy was similar, and showed above-chance classification before critical phoneme onset (green line; orange arrow). Shaded error±s.e.m. across word pairs. (**b**–**e**) Classification weights for all subjects mapped onto a common cortical surface (MNI). During the pre-critical phoneme period, classification performance was driven by bilateral superior temporal cortex for original (**b**) and noise (**c**) trials. Noise trials also showed significantly greater weights in left inferior frontal cortex compared with original trials (orange box). During the post-critical phoneme period, classification performance was driven by bilateral superior temporal cortex for original (**d**) and noise (**e**) trials, with greater weights in left superior temporal cortex for original trials (blue box).

Unexpectedly, we also observed a difference between the two noise percepts that began before the critical phoneme (Supplementary Figs 7 and 8, orange arrow). We can better examine these early brain states using the noise trials alone because each trial is categorized solely according to the listener's subjective perception of the same stimulus. To characterize the spatial and temporal neural activity patterns that give rise to this effect, we applied a single-trial linear classification analysis to quantify the differences in neural activity patterns for each percept (the previous RI analysis was designed to examine the similarity of each noise trial to each original stimulus, and therefore might not be sensitive to non-stimulus-driven differences between responses to the noise stimuli). In line with the PC analysis, we observed that noise trials were classified accurately before critical phoneme onset (one-way *t* test against 50% chance level *t*(5)=2.73, *P*<0.035; Fig. 4a, orange arrow; maximum single subject accuracy=92.7%, occurring 130 ms before critical phoneme onset, and ∼130 ms after word onset, the exact timing of which was variable across word pairs; Supplementary Fig. 9). We only observed this effect for noise trials because the pre-critical phoneme bias is a primary source of information that determines the eventual percept. In contrast, the percept on original trials is largely determined by the acoustic differences between the critical phonemes. Although there are presumably pre-critical phoneme effects for original trials, they are not detectable in the present paradigm since the eventual acoustic cue—typically a much more reliable source of information—will overcome those biases^13^.

This pre-critical phoneme separability of noise trials presumably reflects brain states that bias subsequent perception according to the same phoneme categorization processes that represent the actual sounds after hearing the critical phoneme. We trained a classifier on a 110 ms window around the time when the original stimuli are maximally discriminable, and tested it on all other time points for both original and noise trials. For noise trials, the time period from −300 to −50 ms before the critical phoneme still showed a trend for above-chance classification accuracy (one-way *t* test *t*(5)=2.25, *P*<0.075, uncorrected), although it was less than when separate classifiers were trained over time (Supplementary Fig. 10). This suggests that before speech input, neural states already reflect predictions about upcoming speech sounds^17^, and deeply influence how those sounds are perceived. Pre-stimulus neural bias explains why listeners' perception of noise fluctuates across trials, and furthermore provides evidence for top–down modulation of speech representations^18^,^19^.

To localize the brain areas that were involved in pre-stimulus bias, we mapped the classifier weights on the brain. To determine whether there were significant effects of hemisphere (left, right), location (supra-Sylvian, sub-Sylvian), and condition (original, noise), we ran an ANOVA with these factors across all electrodes for all subjects. There was a significant three-way interaction (*F*(1,516)=6.8, *P*<0.01), allowing us to test specific post-hoc hypotheses. During the pre-critical phoneme time period, both original and noise trials showed strong weights in bilateral STG and medial temporal gyrus (MTG) that were not significantly different across conditions (two-sample *t* test t(60.78)=−2.21, *P*>0.05, Bonferroni corrected for multiple comparisons, *n*=34; Fig. 4b). However, strong weights for noise trials were also observed in left inferior frontal gyrus, pre-central gyrus and post-central gyrus to a greater extent than for original trials (two-sample *t* test *t*(34.52)=−7.40, *P*<10^−7^, Bonferroni corrected for multiple comparisons, *n*=27; Fig. 4c). Frontal neural populations are known to be critical for integrating contextual information^20^, and here we show that these computations can manifest as a pre-stimulus bias that predicts how ambiguous input is subsequently perceived^21^.

After critical phoneme onset, maximal classification accuracy was 75% for original trials and 70% for noise trials (one-way *t* test *t*(5)=13.63, *P*<10^−4^; Fig. 4a, blue arrow). Across participants, the latency of peak accuracy was not significantly different for original and noise trials (paired *t* test *t*(10)=−0.96, *P*=0.36), consistent with the online phoneme restoration effects observed in Fig. 1h. This post-critical phoneme onset restoration effect was mapped primarily to bilateral STG. Classifier weights in left STG and MTG were significantly stronger for original than noise trials (two-sample *t* test *t*(54.70)=4.07, *P*<10^−4^, Bonferroni corrected for multiple comparisons, *n*=34; Fig. 4d,e). This is likely due to auditory neural populations being strongly tuned for spectrotemporal acoustic differences, which are not present in the noise condition^7^.

## Discussion

Together, these results demonstrate that auditory speech circuits in the human brain are remarkably robust to sub-optimal listening conditions, even in cases where the acoustic input is not physically present. The ability to effectively cope with missing acoustic content^22^,^23^,^24^,^25^ is a critical adaptation for effective communication^26^ and for general auditory processing in natural situations. Finding that auditory neural populations are not only robust to complete interruptions of the acoustic signal, but actually generate representations of the missing sound, provides a powerful demonstration of the disconnect between the true nature of sensory input and our perception^27^.

Even under clear listening conditions, the internal dynamics of speech perception networks are highly influenced by predictions related to multiple levels and timescales of linguistic and memory representations^17^,^28^,^29^. Our results suggest a role for left inferior frontal cortex in generating these predictions and bias signals during speech perception. Previous work has demonstrated a top–down modulatory role of these same areas for speech comprehension^14^,^30^,^31^, which is consistent with a network hierarchy that is driven by rapid prediction updating mechanisms^32^. While strongly adaptive for communication, abnormal processing in these same circuits could provide novel mechanistic insights into the perceptual nature of auditory distortions associated with schizophrenia^33^,^34^, where sounds are often misperceived especially in the context of noisy interference.

The unexpected finding of robust pre-critical phoneme classification opens the door for novel investigations of the interactions between internal neural states and bottom-up sensory input. In particular, it will be important to disentangle the independent and interacting contributions of stochastic brain states^13^,^35^,^36^ that reflect ongoing task-irrelevant activity but impact perception, and predictive signals^15^,^29^,^37^, which may be related to learned lexical representations^38^,^39^. Our results do not unambiguously disentangle these two possibilities, however there is evidence that both may deeply influence perception.

Furthermore, it remains unclear whether these pre-critical phoneme bias signals are a prerequisite for perceptual restoration. In our data, there are a small number of examples where classification accuracy before critical phoneme onset is closer to chance, yet subjects still report hearing the missing phoneme, which suggests that the bias signals may simply modulate the extent or speed of restoration. Among the paradigms that may help provide more specific interpretations of the bias signals, previous work has utilized task designs where restored and non-restored trials can be compared directly^24^, and have identified left inferior frontal regions as important in perceptual restoration.

More generally, the observation of a warping of the acoustic-phonetic representation in STG that is preceded by predictive effects in a higher-order cognitive region (left inferior frontal cortex) is inconsistent with models of speech perception that posit post-perceptual decision processes as the locus of restoration^39^. Although we cannot unambiguously identify the nature of these higher-level representations (for example, lexical, semantic and so on), we propose that our results are more consistent with speech perception models that allow for online modulation of perceptual representations by higher-level linguistic information^38^. Ultimately, these findings demonstrate that speech perception at the acoustic level is deeply influenced by neural processes related to prediction and integration of contextual knowledge.

## Methods

### Participants

Five human subjects (4 female, mean age 38.6 years, range 30–47) underwent the placement of a high-density subdural electrode array (4 mm pitch) over the lateral surface of the brain. No subjects had a history of any cognitive deficits that are likely to be relevant to the aims of the present study. All were left hemisphere language-dominant, and all but one were native English speakers (one subject was a native Italian speaker, but was completely fluent in English). Two participants were implanted on the left hemisphere and three were implanted on the right hemisphere. All participants gave written informed consent before surgery, and all protocols were approved by the Committee on Human Research at UCSF.

### Stimuli and tasks

We developed a set of word pairs that allowed us to examine how the brain processes the same acoustic stimulus when it is perceived two different ways. 130 words were selected by searching an online corpus^40^ for words that were five phonemes and two syllables long, and had an English neighbourhood density of one, meaning that replacing a single phoneme in the word creates only one other word in the language (for example, /fæstr/ versus /fæktr/). Stimuli were synthesized using the built-in ‘Alex' voice in Mac OSX. To create the restoration stimuli, the critical phoneme was identified in Praat^41^, and it was excised along with surrounding coarticulatory cues. The silent gap was then filled with 1/*f* noise that was matched to the root-mean-square amplitude of the phoneme it replaced to provide a plausible masker for restoration. Six subjects participated in a pilot study modelled on previous phoneme restoration behavioural studies^2^,^3^ where they listened to these stimuli and indicated what word they heard, how confident they were in their response, and which phoneme had been replaced. Twenty word pairs with high confidence ratings and a range of accuracy on replaced phoneme identification were selected for the neural study. We specifically selected stimuli where pilot subjects heard the noise as both possible words, however, we also included word pairs that had particularly strong confidence ratings, even though only one alternative was heard. Since the strength of perceptual restoration can vary within individuals, we presented ECoG participants with this larger set of words to ensure that we would capture examples where they experienced bistable perception.

Three participants (two left hemisphere and one right hemisphere) completed 10–12 blocks of the single word repetition task, where they heard each of the three versions of the 20 words (for example, /fæstr/, /fæktr/, /fæ#tr/) in a random order within each block, and were asked to repeat what they heard on each trial. After each production, the experimenter advanced to the next trial, meaning that there was a variable inter-stimulus interval. For the right hemisphere patient, 10 stimuli (appoint/anoint, ethics/epics, option/auction, proper/proffer, safety/safely, service/nervous, sorrows/borrows, torture/torpor, waters/walkers and woven/woken) were removed from the task, since they did not produce bistable perception, or the originals were not perceived correctly in the first two patients (two stimuli were added for this patient: menu/venue and engage/enrage). For all patients, other stimuli were excluded from analysis if listeners did not correctly perceive the originals. There were only a small number of cases in which the originals were not perceived correctly, mostly due to difficulty in splicing sounds with unnatural coarticulatory cues, or the relative low lexical frequency of one of the words. Two right hemisphere participants completed 3–6 blocks of the sentence context task, where four word triplets were preceded by semantically congruous (‘On the highway he drives the car much *faster*'), incongruous (‘On the highway he drives the car much *factor*') or biased (‘On the highway he drives the car much [/fæ#tr/]') frames (three repetitions of each critical word in each context per block). They were asked to indicate with a button press whether the sentence made sense or not (note that it is possible for listeners to rationalize some incongruous sentence meanings, which may make the task somewhat difficult). We did not observe any appreciable differences in either the behavioural or neural results between tasks, and therefore included data from both tasks in all analyses. However, we present examples from each task separately (for example, Fig. 1 versus Supplementary Fig. 1; Fig. 3 versus Supplementary Fig. 2) to demonstrate the similar effects observed. There were four word pairs on which subjects showed bistable perception for the noise stimuli, defined as perceiving the noise as each of the possible words on at least 25% of trials (Supplementary Table 1). Other word pairs did not yield bistable perception, but still produced robust neural phoneme restoration effects consistent with participants' reported perception on each trial (Fig. 2). Every participant showed behavioural and neural phoneme restoration effects (including bistable perception).

### Data pre-processing

Electrocorticography was recorded with a multichannel amplifier optically connected to a digital signal processor (TuckerDavis Technologies). Channels and time segments containing artifacts or excessive noise were removed before a common average reference across rows of the 16 × 16 electrode grid. Grid placement was determined solely by clinical considerations, and typically did not include more dorsal or anterior prefrontal regions. The high-gamma (70–150 Hz) analytic amplitude was extracted using previously published procedures^7^, and was *z*-scored relative to a 500 ms pre-stimulus window. Each trial was time-locked to the acoustic onset of the word. Since the measures of neural phoneme restoration were based on reliable differences between the original stimuli in a word pair, we selected electrodes that distinguished those stimuli using *z*-score thresholds for the difference between originals. These *z*-scores varied across subjects between 1 and 2.25 (however, see below for confirmation that these thresholds are not critical for the observed effects). Electrodes that showed these differences during a time window from the onset of the critical phoneme to the offset of the word were included in subsequent analyses. This resulted in 6–56 electrodes per word pair (Supplementary Table 1).

To ensure that the results were not an artifact of the electrode selection process, we performed each analysis with two alternative electrode sets: (1) A less stringent criterion for which electrodes showed differences between originals, and (2) All electrodes on the ECoG grid. In all cases, results were qualitatively similar, therefore we present the data from electrodes that showed the greatest differences between originals. We also confirmed our results using anatomical region-of-interest analyses. For example, we demonstrated that spectrogram reconstruction was similar when using selective electrodes versus all electrodes over STG (but not frontal cortex electrodes; Supplementary Figs 4 and 5; note that this includes all supra-Sylvian electrodes). We ultimately chose to be largely agnostic to functional anatomy priors because recent results demonstrate that non-auditory cortical regions are selective to acoustic properties^42^, and functional clustering of mesoscopic neural signals often outperforms anatomical region-of-interest approaches^43^,^44^,^45^.

For single electrode analyses, phoneme restoration effects were quantified in the high-gamma evoked responses by calculating 99% confidence intervals with a bootstrapping procedure with 1,000 randomizations. Time points that satisfied the following confidence interval criteria were considered to show significant phoneme restoration effects:

1. Orig1≠Orig2
2. (Orig1=Noise1) & (Orig1≠Noise2)
3. (Orig2=Noise2) & (Orig2≠Noise1)

### Restoration index

To quantify the degree of neural phoneme restoration on each electrode, we defined the RI as:


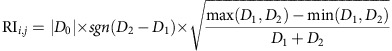


where the RI on electrode *i* at time point *j* is a function of the relative distance between the noise stimulus response and the two original stimulus responses. *D*_1_ is the Euclidean distance between the noise and original 1, and *D*_2_ is the distance between the noise and original 2. *D*_0_ is the distance between the two originals. RI values were calculated on each electrode at each time point for each percept. The sign of the RI is arbitrary, however its definition ensures that positive values reflect the similarity of the response to noise with one original word, and negative with the other original word. This means that each percept in a bistable word pair is ultimately assigned an arbitrary sign. Therefore, to combine data across word pairs, we calculated the magnitude of the difference between RI values for a given word pair. For word pairs that did not produce bistable perception, positive RI values were arbitrarily assigned to the word participants perceived.

### Stimulus spectrogram reconstruction

We used a decoding method that calculates a linear mapping between electrode population neural activity and the acoustic spectrogram of each stimulus^46^. Briefly, this method relates a neural response *R* on electrode *n* at time *t* to the stimulus spectrogram *S* at time *t* and frequency band *f* (ref. 11):


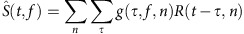


Reconstruction filters were learned from data collected during separate testing sessions for each subject from a natural speech corpus^47^ with filter time lags from -300 to 0 ms. These filters were then fit to the phoneme restoration word triplets to obtain reconstructed spectrograms. Reconstructions were performed on the mean of the trials for each condition, however similarly robust results were observed with single trial analyses. To quantify the fit of the reconstructions, 2D correlations were calculated between all pairwise combinations of original and noise stimuli. Statistical comparisons on the power spectra of the critical phonemes were calculated using the frequency bands that best discriminated the two original stimulus spectrograms (90th percentile of frequencies), and computing the Euclidean distance between those frequencies for each condition. Since filters were derived from passive listening to natural speech, the reconstructions are not related to explicit phoneme decisions, but instead reflect the spectrotemporal sensitivities of underlying neural populations.

### Neural ‘state-space' analysis

To visualize neural activity patterns across electrodes, we used principal component analysis, an unsupervised dimensionality reduction method^16^. For each subject, the data were reshaped into an *n* time points x trials by *p* electrodes matrix. The PCs represent optimal linear combinations of electrodes along a set of orthogonal bases, and therefore describe the variance across the neural population. Plotting the activity of each PC against time or multiple PCs against each other provides visualizations of trajectories through this lower-dimensional space as each stimulus is processed. To determine the contributions of each electrode to each PC, we plotted the PC weights on the brain.

### Stimulus classification

A series of linear support vector machines were trained to classify the participant's reported perception (chance=50%) from the neural population responses. Due to relatively small sample sizes, data were augmented at each time point by concatenating neural responses from a sliding symmetric 110 ms time window. Leave-one-out cross validation was used for each classifier to prevent over-fitting. Separate classifiers were trained and tested on original and noise trials, however, identical parameters were used in all cases. To determine which brain regions contributed to classification, the squared beta values from Matlab's *fitcsvm* function were plotted on the cortical surface across subjects and word pairs using a 4.5 cm Gaussian smoothing kernel. These values are analogous to weights in a linear regression model, describing the relative impact of each electrode on the definition of the support vectors. Weights were statistically compared across conditions during the pre- and post- critical phoneme time windows using independent samples *t* tests with Bonferoni corrected *P* values.

### Code availability

All analyses were performed using Matlab R2014b, with standard functions and toolboxes. All code is available upon request.

### Data availability

The data that support the findings of this study are available from the corresponding author upon reasonable request.

## Additional information

**How to cite this article:** Leonard, M. K. *et al*. Perceptual restoration of masked speech in human cortex. *Nat. Commun.* **7,** 13619 doi: 10.1038/ncomms13619 (2016).

**Publisher's note:** Springer Nature remains neutral with regard to jurisdictional claims in published maps and institutional affiliations.

## Supplementary Material

Supplementary Information Supplementary Figures 1-10 and Supplementary Table 1.
