# Supplementary material for: Perceptual restoration of masked speech in human cortex
Source: Nat Commun. 2016 Dec 20;7:13619. doi: 10.1038/ncomms13619 (PMC5187421; doi:10.1038/ncomms13619)
Supplement: Supplementary Information — Supplementary Figures 1-10 and Supplementary Table 1. [file ncomms13619-s1.pdf]

Supplementary Figures

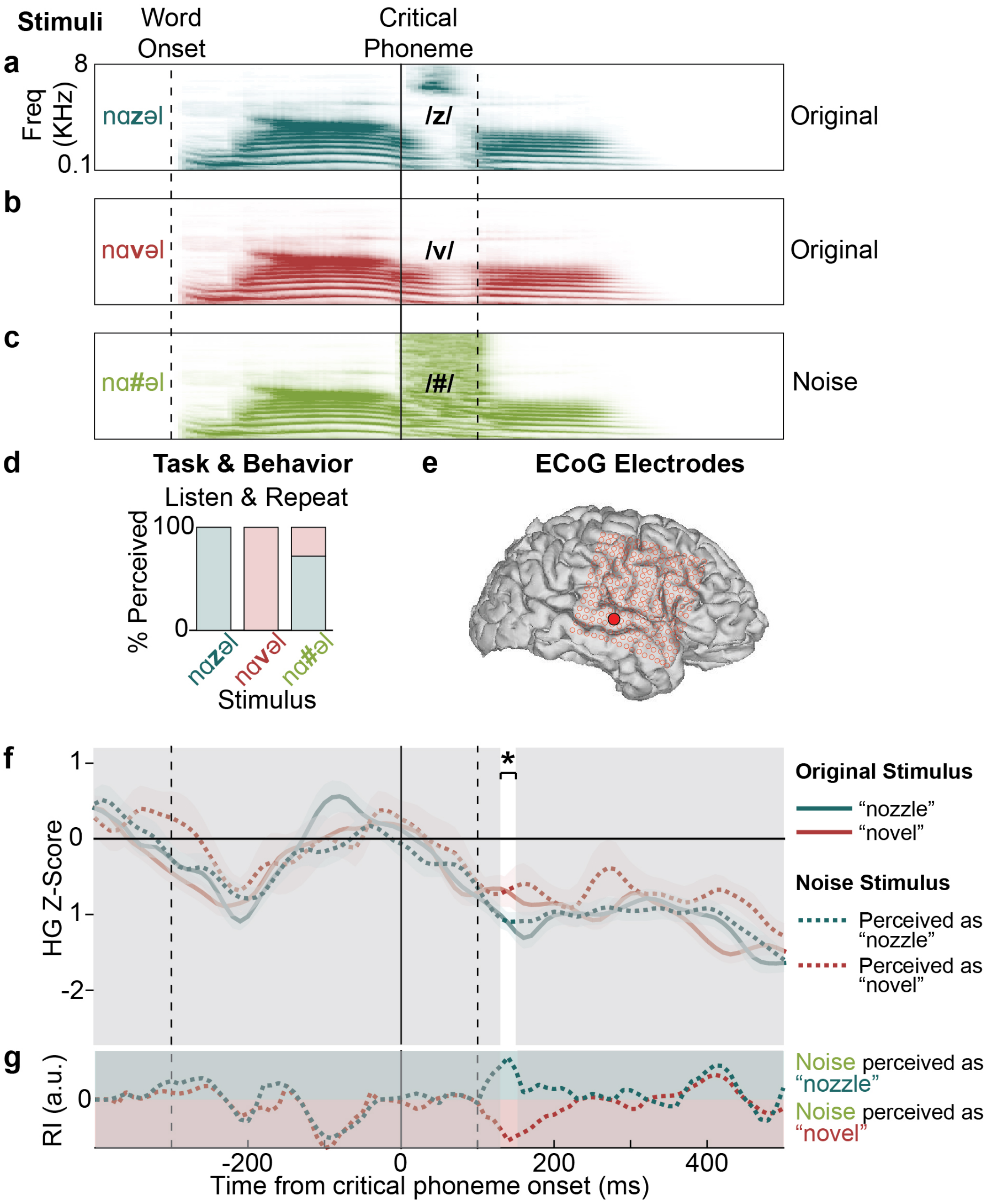

Supplementary Fig 1: Stimuli and single electrode online phoneme restoration effects from the sentence task. a,b, Subjects listened to pairs of spoken words (/nɑzəl/ (**a**) vs. /nɑvəl/ (**b**)) that were acoustically identical except for a critical phoneme that differentiated their meaning (vertical solid and second dashed lines; first dashed line is word onset). **c,** The critical phoneme was also replaced by broadband noise (/nɑ#əl/), and on each trial subjects reported which word they heard. **d,** Behavioral results show bistable perception on noise trials. **e,** Location of representative posterior STG electrode in (**f**). **f,** Single representative right hemisphere STG electrode shows selectivity for /z/ compared to /v/ (solid blue line stronger response than solid red line immediately after critical phoneme, unshaded region). Trials were sorted depending upon which word participants perceived. Responses to noise stimuli were similar to the original version of the perceived phoneme (dotted lines; *: 99% CIs only overlapping for same colored curves; shaded error ± s.e.m. across trials). This word pair occurred in the sentence context task, but showed responses that were similar to those observed in the single word task. **g,** Restoration Index (RI) describes the magnitude of neural restoration as the relative distances between each noise and original pair in (**b**). When the dotted line is in the region shaded with the same color, the electrode's activity reflects the subject's percept.

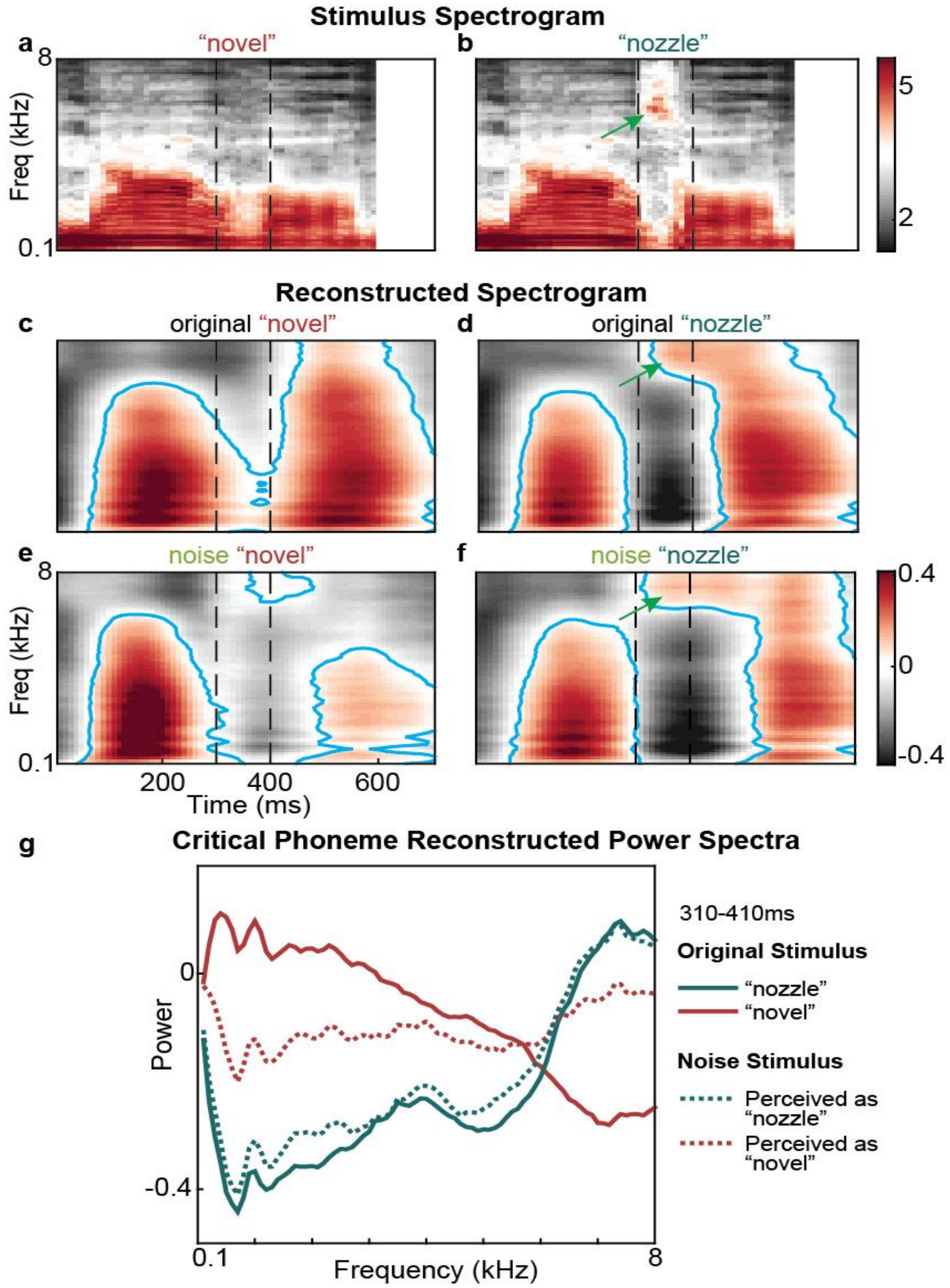

Supplementary Fig. 2: Stimulus spectrogram reconstruction reveals warping of noise to perceived phoneme in the sentence task. a,b, Acoustic spectrograms for a representative word pair (/nɑvəl/, **a**, vs. /nɑzəl/, **b**) differ primarily on the presence of a high frequency component during the critical phoneme in **b** (green arrow). The words followed biasing sentence contexts. **c,d,** Spectrograms from (A,B) reconstructed from electrode population activity show that the high frequency component is present in /nɑzəl/ (**d**, green arrow) and absent in /nɑvəl/ (**c**). **e,f,** Spectrogram reconstruction of noise trials was divided according to which word the participant heard on each trial. During the critical phoneme, a high frequency component is visible only for trials perceived as /nɑzəl/ (**f**, green arrow) and not for /nɑvəl/ (**e**). Both original and noise /nɑzəl/ also show similar decreases in acoustic power in lower frequencies compared to /nɑvəl/. **g,** Reconstructed power spectra of the critical phoneme for **c-f** reveal close correspondence between noise and original phonemes, particularly in mid-high frequencies.

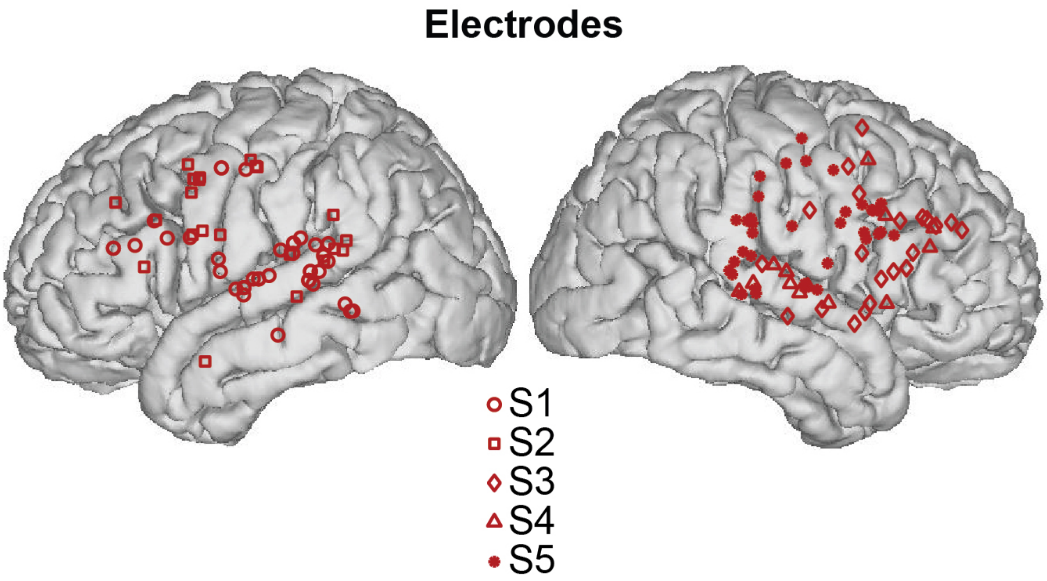

Supplementary Fig. 3: Electrodes selected for population analyses and spectrogram reconstruction. Across all participants and word pairs, 131 bilateral temporal and frontal electrodes discriminated original words during the time window from critical phoneme onset to word offset. This criterion was used to select electrodes because our measure of neural phoneme restoration (quantified in the RI metric; see **Fig. 1g-h**, **Methods**) is most sensitive when there is a discernible difference between the originals.

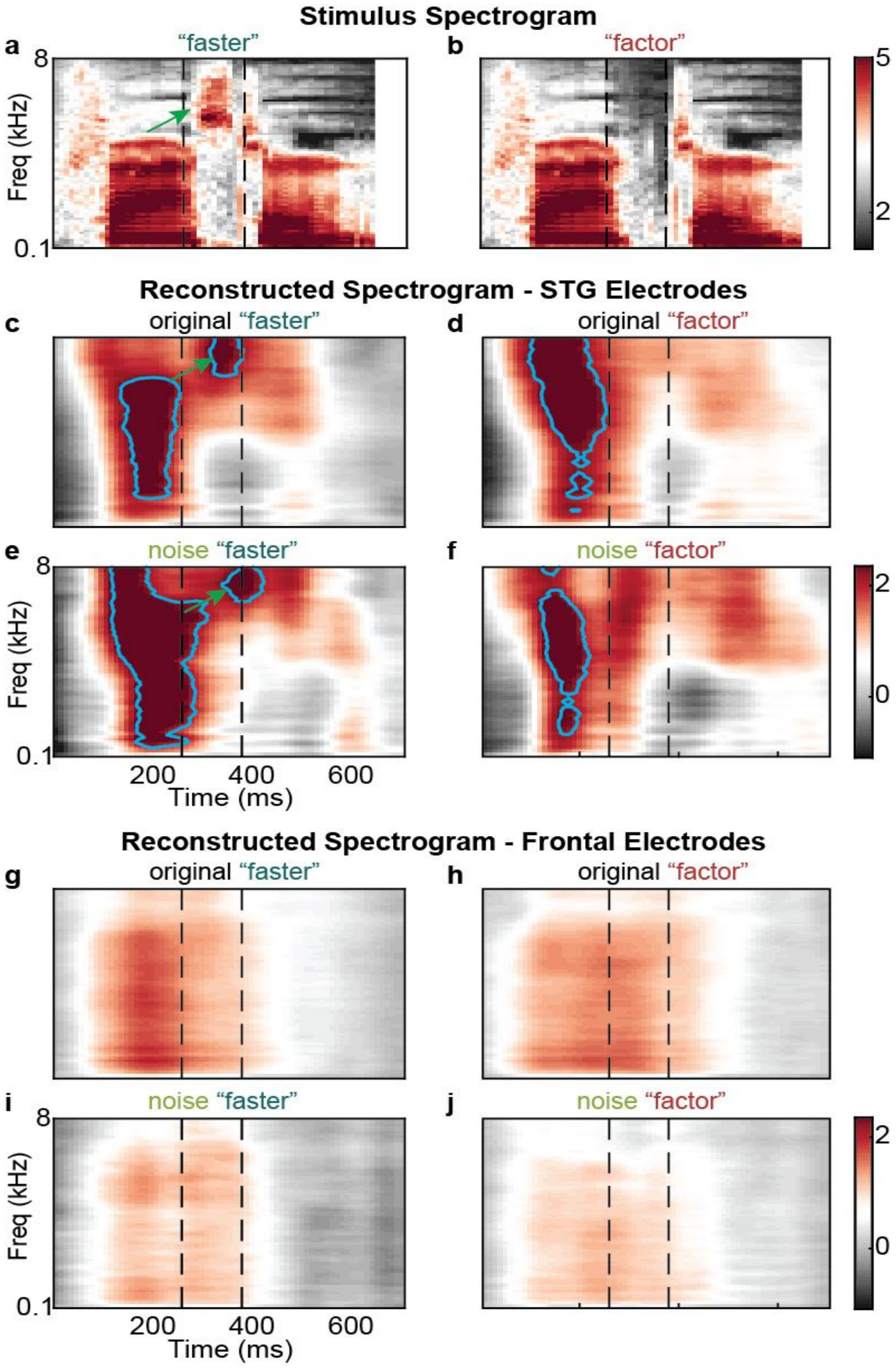

Supplementary Fig. 4: Stimulus spectrogram reconstruction in auditory and frontal electrodes. a,b, Acoustic spectrograms for a representative word pair (/fæstr/, **a**, vs. /fæktr/, **b**) differ primarily in the presence of a high frequency component during the critical phoneme in **a** (green arrow). **c-f,** Spectrograms from (A,B) reconstructed from STG electrode population activity (defined by an anatomical atlas) show that the high frequency component is present in /fæstr/ (**c**, green arrow) and absent in /fæktr/ (**d**). Spectrogram reconstruction of noise trials was divided according to which word the participant heard on each trial. During the critical phoneme, a high frequency component is visible only for trials perceived as /fæstr/ (**e**, green arrow) and not for /fæktr/ (**f**). **g-j,** Spectrograms from (A,B) reconstructed from frontal electrode population activity (defined by an anatomical atlas) show that discriminative stimulus features are not reconstructed.

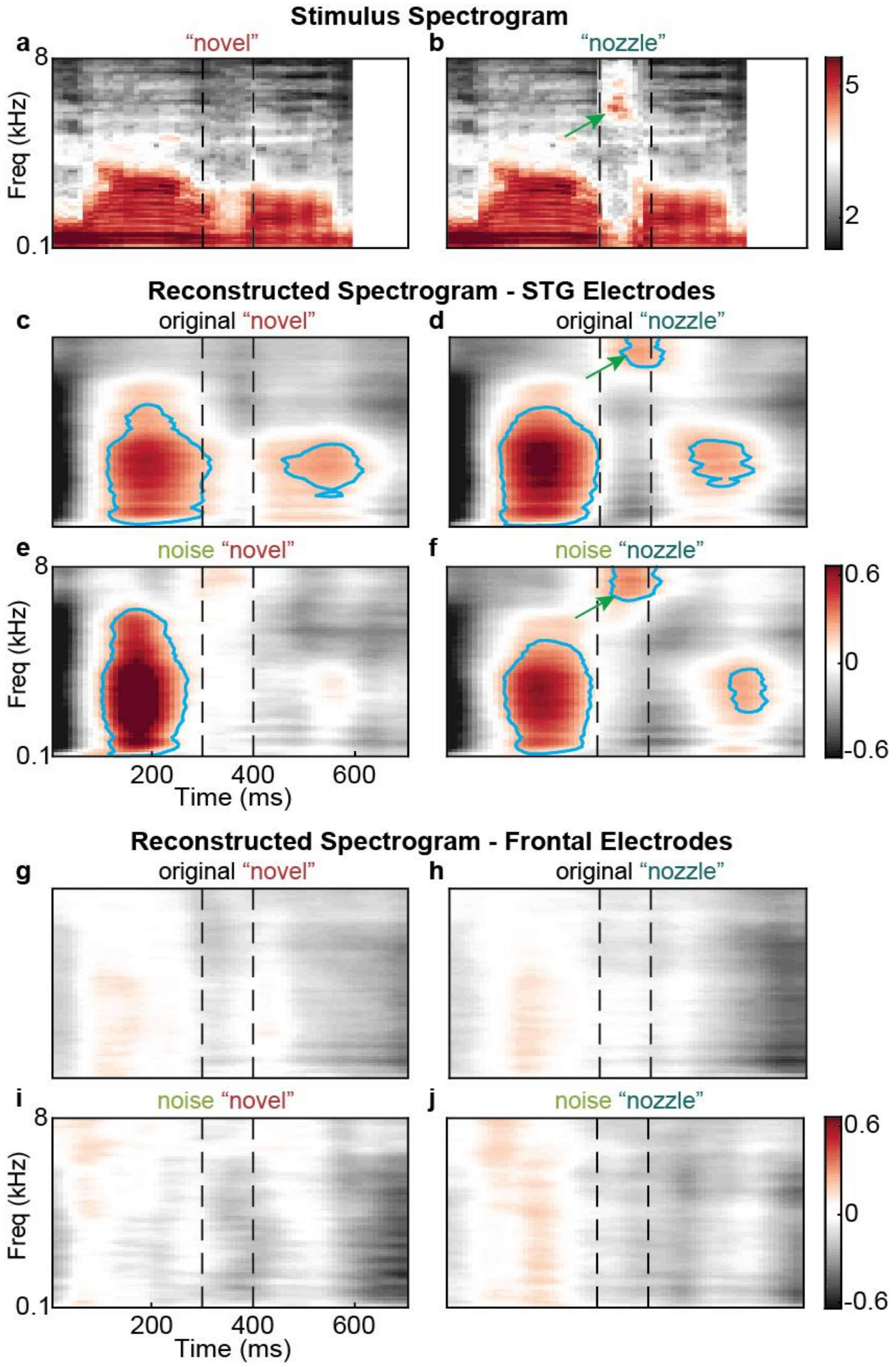

Supplementary Fig. 5: Stimulus spectrogram reconstruction in auditory and frontal electrodes from the sentence task. a,b, Acoustic spectrograms for a representative word pair (/nɑvəl/, **a**, vs. /nɑzəl/, **b**) differ primarily in the presence of a high frequency component during the critical phoneme in **a** (green arrow). **c-f,** Spectrograms from (A,B) reconstructed from STG electrode population activity (defined by an anatomical atlas) show that the high frequency component is present in /nɑzəl/ (**c**, green arrow) and absent in /nɑvəl/ (**d**). Spectrogram reconstruction of noise trials was divided according to which word the participant heard on each trial. During the critical phoneme, a high frequency component is visible only for trials perceived as /nɑzəl/ (**e**, green arrow) and not for /nɑvəl/ (**f**). **g-j,** Spectrograms from (A,B) reconstructed from frontal electrode population activity (defined by an anatomical atlas) show that discriminative stimulus features are not reconstructed.

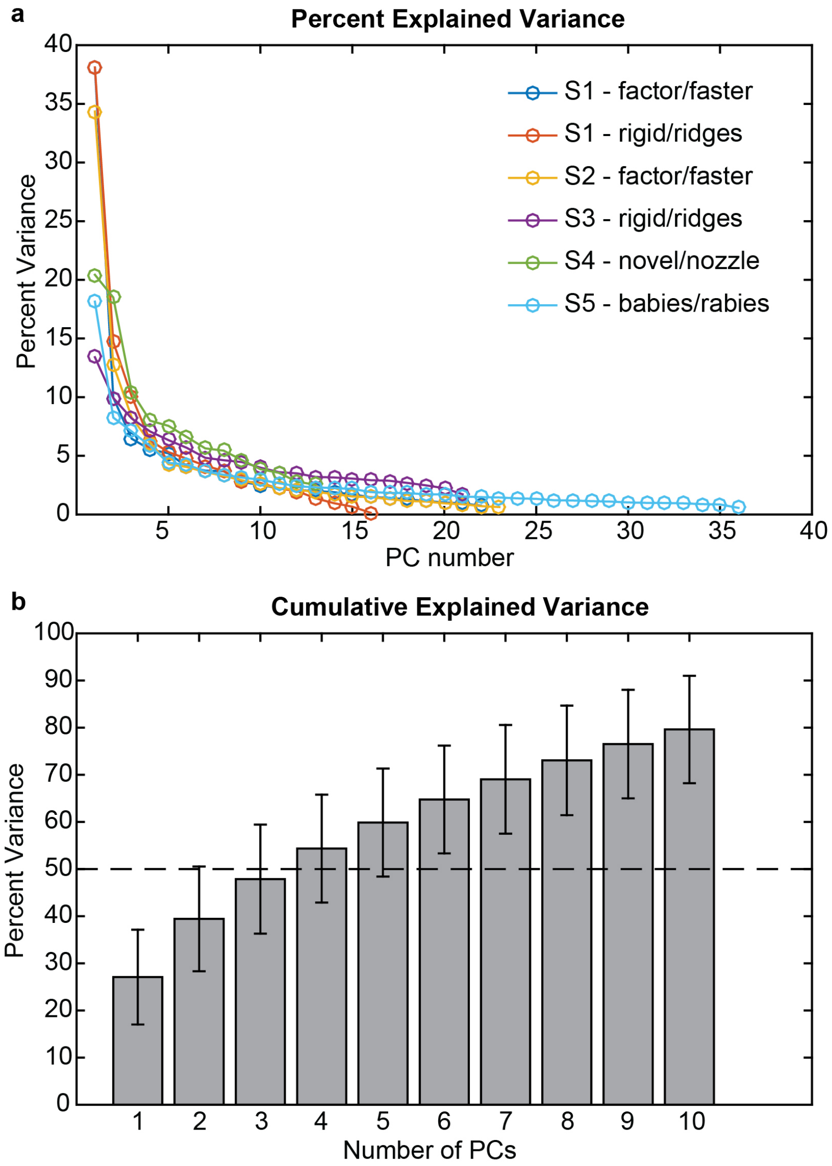

Supplementary Fig. 6: Percent explained variance for single trial population state-space analyses using PCA. a, Percent variance is shown as a function of the PC number for each subject/word pair (colored lines). All curves are similar, and show an 'elbow' around 4-5 PCs. PCs are linear combinations of electrodes, explaining the shared variance across spatial patterns of neural activity. **b,** Cumulative explained variance shows that 4-5 PCs are sufficient to describe 50% of the variance across electrodes. Error bars represent s.d. across subjects/word pairs.

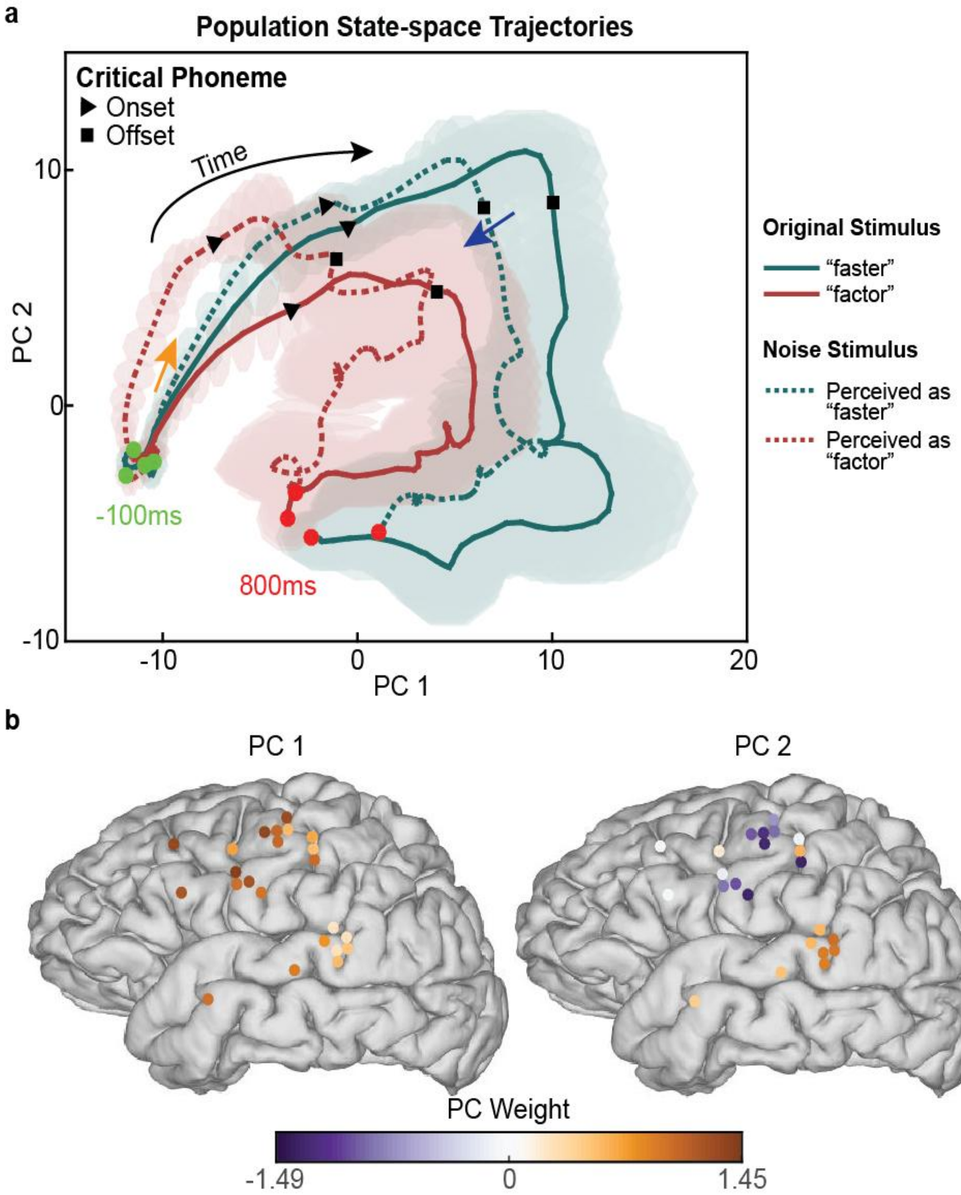

Supplementary Figure 7: Single trial neural state-space analysis. a. Neural 'state-space' visualization (one participant) summarizes population activity patterns across time as trajectories through a 2-dimensional space. Consistent with RI plot in **Fig. 1h**, peak perceptual phoneme restoration effects occur just after critical phoneme offset (blue arrow). Noise trials also differentiate immediately after word onset (orange arrow), indicating pre-critical phoneme predictive influences on perception. **b.** PC weights plotted on the brain for PCs 1 and 2. PC 1 shows the strongest weights in frontal and anterior regions, while PC 2 shows the strongest positive weights in posterior STG. In the state-space visualization, PC 1 shows the separation between the two noise percepts immediately after stimulus onset (orange arrow), while PC 2 shows the separation between the perception of the two possible words (blue arrow).

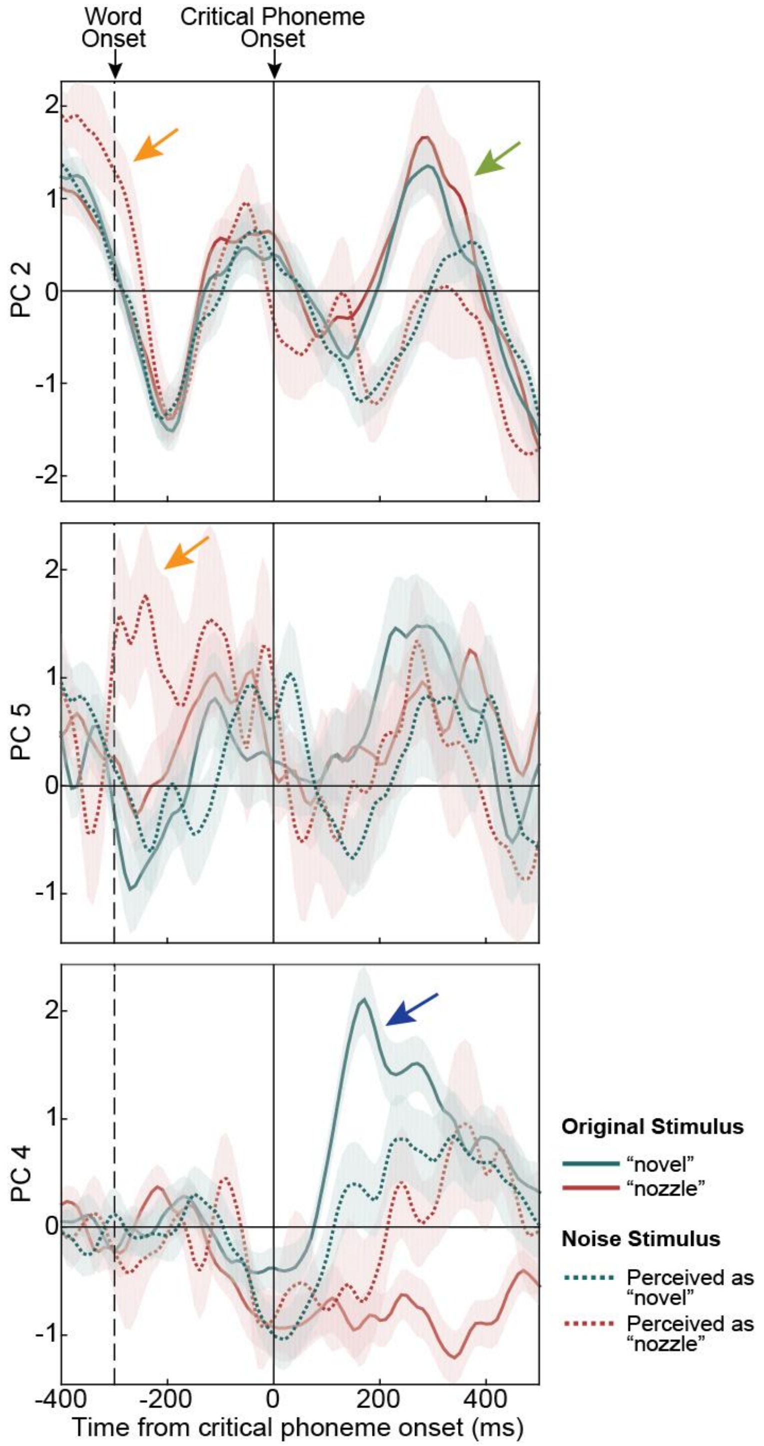

Supplementary Fig. 8: Single trial neural state-space analysis shows neural phoneme restoration effects and pre-stimulus bias. PCA was performed across electrodes for "novel/nozzle" for a participant who completed the sentence context task. PCs 2, 4, and 5 represent linear combinations of activity across electrodes that illustrate important phoneme restoration phenomena. In PC 2, there is a clear pre-critical phoneme difference between noise stimuli (dotted lines) that is not observed for original stimuli (solid lines; orange arrow), similar to **Supplementary Figure 7**. PC 2 also shows evidence of noise encoding at ~200ms post-critical phoneme onset (green arrow). PC 5 shows a similar pre-critical phoneme bias for noise trials. Finally, PC 4 captures variance that explains the online restoration effect, where noise trials are similar to their analogous original trials (blue arrow).

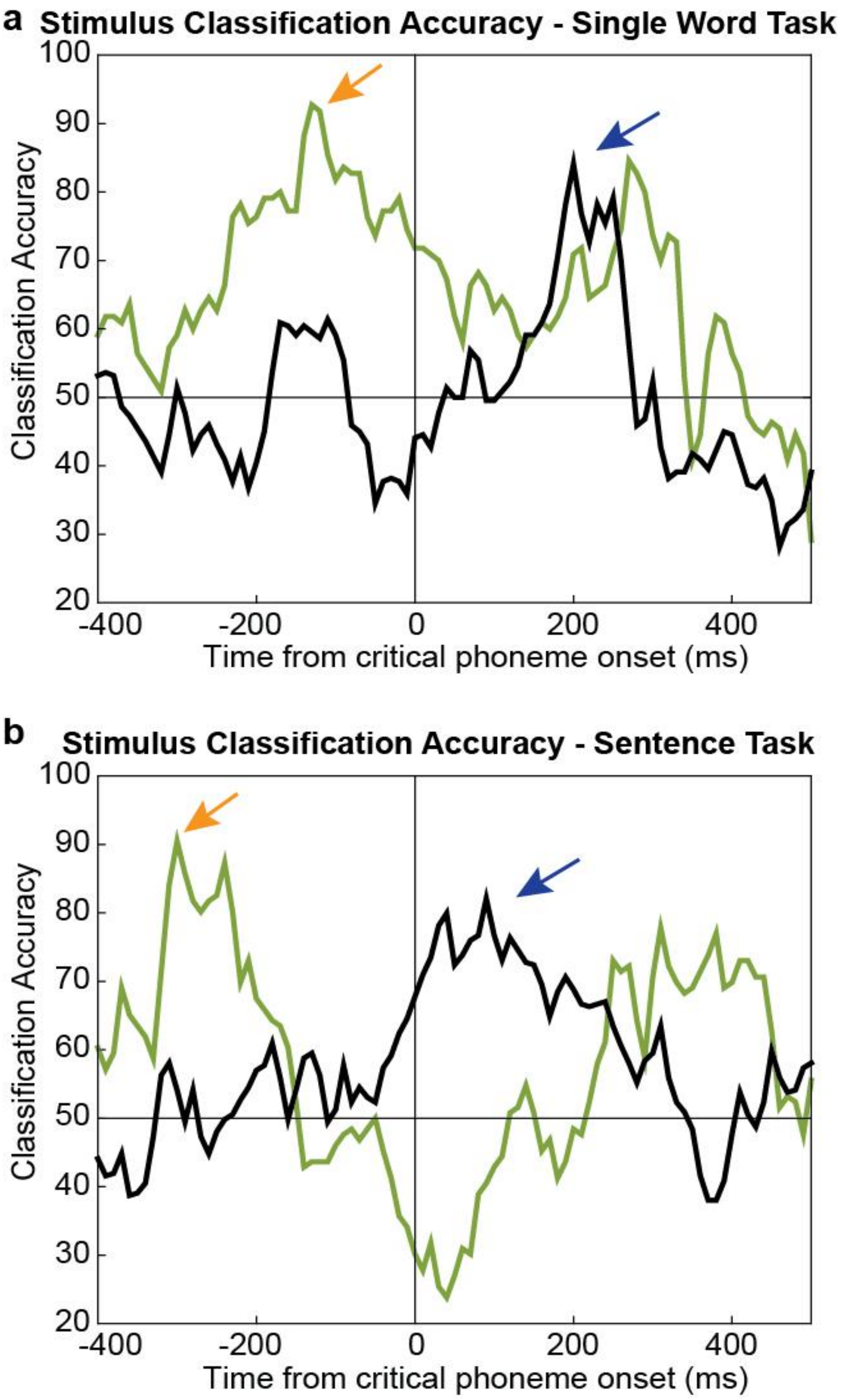

Supplementary Fig. 9: Timecourse of stimulus classification shows pre-critical phoneme bias for noise phonemes in representative participants. a. In the single word task, classification accuracy for both original (black line) and noise (green line) trials reached ~85% at ~200ms post-critical phoneme onset (blue arrow). Additionally, noise trials were classified correctly 92.7% of the time ~130ms before critical phoneme onset (orange arrow), illustrating pre-critical phoneme neural bias. **b**. Similar results were observed in the sentence context task.

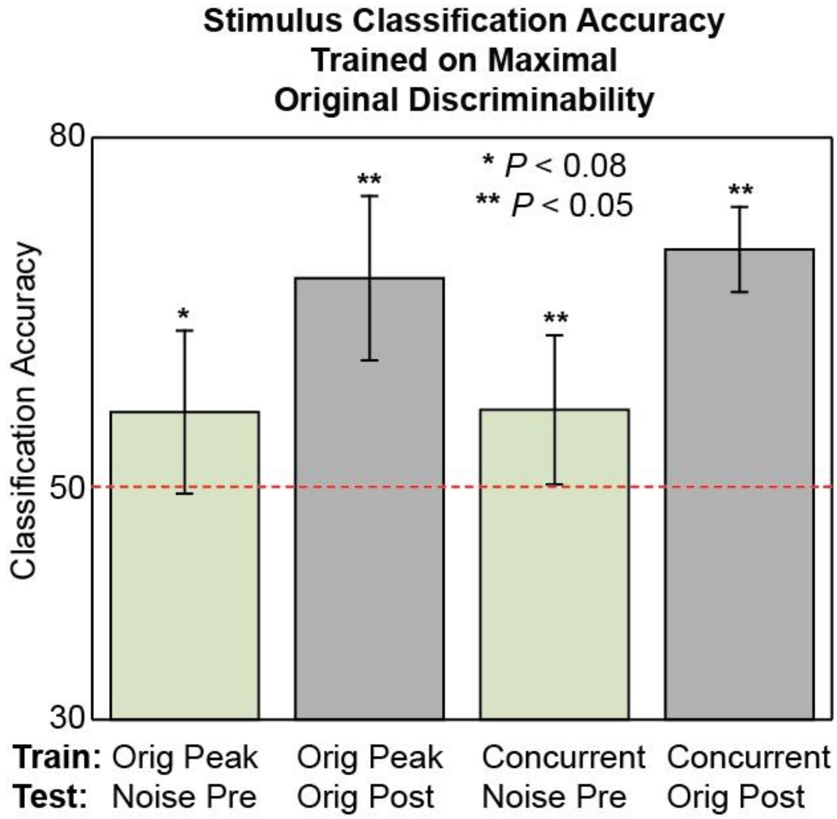

Supplementary Fig. 10: Pre-critical phoneme bias for noise trials partially reflects acoustic discriminability. The classifier was trained on the time window where the original stimuli are maximally discriminable (**Fig. 4a**, blue arrow). To determine whether the pre-stimulus classification results for noise trials reflect representations of the acoustic-phonetic differences observed for the original stimuli, we tested the classifier on time points before the critical phoneme. Classification accuracy had a trend for significance above chance (one-way *t*-test $P = 0.075$; first green bar), compared to significantly above chance accuracy when the classifier was trained and tested on concurrent timepoints (one-way *t*-test $P < 0.035$; second green bar; **Fig. 4a**). For original stimuli, classification accuracy was significantly above chance for the classifier trained at the post-critical phoneme peak (one-way *t*-test $P < 0.002$; first black bar), and for the classifier that was trained and tested on concurrent timepoints (one-way *t*-test $P < 10^{-4}$; second black bar). Therefore, above-chance classification accuracy for noise trials before critical phoneme onset is partially due to the fact that the brain is in a state that biases subsequent perception toward one or the other percept of the acoustic-phonetic input.

Supplementary Table 1: Stimulus pairs eliciting bistable perception. Bold rows indicate stimuli with bistable perception.

Participant	Word 1	Word 2	Perceptual Split (%)	# of Electrodes
**1**	**factor**	**faster**	**67/33**	**22**
**1**	**rigid**	**ridges**	**40/60**	**16**
1	listen	linen	100/0	44
1	appoint	anoint	100/0	25
1	cassette	cadet	100/0	53
1	option	auction	100/0	39
1	outside	outsize	100/0	6
1	python	pylon	100/0	42
1	safety	safely	100/0	52
1	service	nervous	100/0	41
1	sorrows	borrows	100/0	38
1	typhoon	tycoon	100/0	47
1	voices	choices	100/0	28
1	waters	walkers	100/0	34
1	woven	woken	100/0	56
**2**	**factor**	**faster**	**40/60**	**23**
2	listen	linen	100/0	26
2	cassette	cadet	100/0	38
2	proper	proffer	100/0	25
2	python	pylon	100/0	26
2	babies	rabies	100/0	43
2	service	nervous	100/0	45
2	sorrows	borrows	100/0	38
2	torture	torpor	100/0	37
2	typhoon	tycoon	100/0	27
2	waters	walkers	100/0	12

**3**	**rigid**	**ridges**	**29/71**	**21**
**4**	**novel**	**nozzle**	**28/72**	**13**
**5**	**babies**	**rabies**	**67/33**	**36**
5	listen	linen	100/0	33
5	faster	factor	100/0	23
5	novel	nozzle	100/0	27
5	outside	outsize	100/0	15
